# A meta-analysis: the efficacy and effectiveness of polypeptide vaccines protect pigs from foot and mouth disease

**DOI:** 10.1038/s41598-022-26462-x

**Published:** 2022-12-19

**Authors:** Jiao Jiao, Peng Wu

**Affiliations:** College of Life Sciences, Shihezi University, Shihezi, China

**Keywords:** Vaccines, Infection

## Abstract

The protective effects of peptides on pigs are controversial. In this study, meta-analysis was used to analyze the protective immune response of peptides. The China National Knowledge Infrastructure, PubMed, Wanfang Data, Cochrane Library, Embase, and gray literature sources were searched for FMDV articles published from the inception of the databases to March 2022. Of the 1403 articles obtained, 14 were selected using inclusion criteria. The experimental data on polypeptide vaccines were analyzed using Microsoft Office Home and Student 2019 Software. From the results, polypeptide vaccine doses (PPVDs) ≤ 1 mg offered protection against FMDV in 69.41% pigs lower than World Organization for Animal Health (OIE) standard (75%, 12/16). PPVDs ≥ 2 mg provided protection against FMDV in 97.22% pigs. When the two groups were compared directly, PPVDs ≥ 2 mg (93.75%) was higher than PPVDs ≤ 1 mg (63.16%). PPVDs ≤ 1 mg provided protection 56% pigs and the inactivated vaccine was 93.33% in direct comparison. In conclusion, PPVDs has a dose-dependent protective effect on pigs and PPVDs ≤ 1 mg group was lower than the inactivated vaccines group.

## Introduction

Foot and mouth disease virus (FMDV) belongs to the family picornaviridae, and is a single-stranded positive-sense RNA virus of the genus Aphthovirus^1^. Foot and mouth disease (FMD) has caused severe economic losses to millions of farmers worldwide^2^. The World Organization for Animal Health lists FMD as a class A animal infectious disease. FMD vaccination reduced the number of animals suffering from clinical disease, virus replication, and persistent infection. The peptide corresponding to the major immunogenic site of VP-1 triggers a protective neutralizing antibody reaction in cattle and pigs^3^. The immunogenicity of the neutralizing antibody of FMDV is contained in amino acid positions 135–160 and 200–213^4^. The highly effective FMDV recombinant epitope vaccine should be similar to the natural VP1 of FMDV^5^. Some antibodies only interact with the G-H loop of VP1, and rarely make other contacts with the capsid of the virus^6^.

Even in the same article, the immune protection dose of polypeptides was inconsistent. Maprianova (2000) showed that 0.5 mg antigen payload protected zero animals, while 2 mg antigen payload protected all the animals^7^. Chan (2001) showed that 0.5 mg antigen payload protected 2/5 of animals, and 2 mg protected all animals^8^. Yang (2007) showed that 0.5 mg and 5 mg could protect all animals^9^. Cañas-Arranz (2019) also proved that 0.5 mg and 2 mg protected as many animals^10^. Hyun (2021) completely protected the animals with a dose of 0.15 mg^11^.

In this study, meta-analysis was used to determine the protective immune response of polypeptides vaccines and clarify the influence of dose on protective effect. To protect the welfare of more animals, all the animal experiments in this study have been published^12^. All the animal experiments in this study have been published to determine the protective immune response of polypeptides vaccines by the help of meta-analysis or met statistical approach. In addition, it increases the statistical efficiency, which a single experiment does not have, and summarizes the existing data.

## Methods

### Literature search strategy

For the meta-analysis, two researchers searched the databases of the China National Knowledge Infrastructure, PubMed, Wanfang Data, Cochrane Library, Embase, and gray literature sources for FMDV literature published from the inception of the databases to March 2022. The keywords used are as follows: “FMDV”, “vaccine”, “pig”, and “swine”.

### Inclusion and exclusion criteria

The inclusion criteria were as follows: ① published Chinese or English literature on FMDV vaccines; ② the same article contains the effectiveness of efficacy experiments of pigs; ③ literature including the challenge of FMDV; ④ sufficient number of animals for data extraction. ⑤ The vaccines included polypeptide vaccines and polypeptides included different expression vectors.

The exclusion criteria were as follows: ① references to the literature reviewed; ② no pigs in the literature; ③ replicated data; ④ lack of data extraction; ⑤ unavailability of full text; ⑥ no inclusion of genetic vaccines, such as DNA vaccines and adenovirus vaccines; ⑦ no inclusion of whole virus inactivated vaccines.

### Data extraction

Two researchers (PW and JJ) conducted a preliminary screening by reading the titles and abstracts. Based on the inclusion and exclusion criteria, the full text and selected articles were read for further analysis. Different opinions were settled through discussions. The data were extracted independently, and input into a specially designed data extraction table. This database was built using Microsoft Office Home and Student 2019 Software.

### Data handling

For a protective immune response of polypeptide vaccines, the high dose group and low dose groups were separated. PPVDs was divided into two groups: the less than or equal to 1 mg group and more than or equal to 2 mg group. In order to study the protective effects of low-dose group, the first analysis was conducted directly, and compared PPVDs less than or equal to 1 mg with that of non-immune group. In order to study the protective effects of the high-dose group, a second analysis was made with PPVDs more than or equal to 2 mg group and the non-immune group. In order to directly compare the difference between the group less than or equal to 1 mg and the group greater than or equal to 2 mg, the third analysis was conducted. Finally, the group less than or equal to 1 mg was compared with the inactivated vaccine group directly. All control group and experimental groups were from the same study. analyses were performed using Microsoft Office Home and Student 2019 Software.

## Results

### Literature screening results

**Figure 1:**
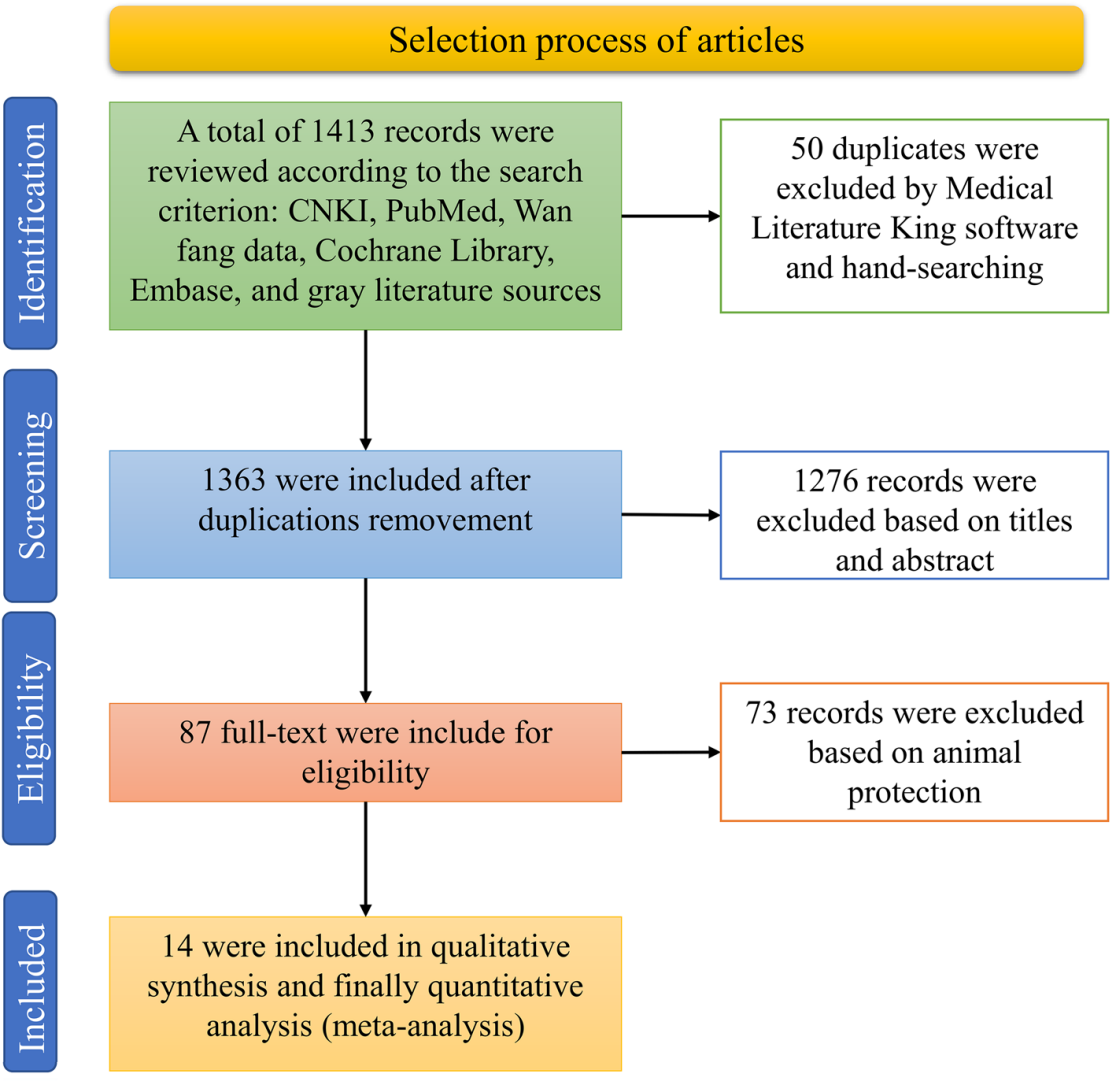
PRISMA flow diagram of search results.

By searching the databases of the CNKI, PubMed, Wanfang data, Cochrane Library, Embase, and gray literature sources, a total of 1413 articles on FMDV were searched. After deleting 50 duplicate articles and reviewing the titles and abstracts, 87 articles were found to meet the inclusion criteria (Fig. 1). Finally, 14 articles were selected for the meta-analysis.

### Experimental data extraction

Table 1 shows the features of the selected studies. A total of 197 animals were involved. All the studies were carried out between 2000 and 2021. The types of polypeptide vaccines used were synthetic, prokaryotic expression, and plant expression based. The injection mode was mainly intramuscular injection, though the injection sites were different. Only one group received the vaccine via the oral route. The immunization dose was 0.015–7.000 mg.

**Table 1:** Characteristics and summary findings of the selected studies^2,8–11,13–20^.

	Author	Year	Protected number	Total number	Type	Expression vector	Vaccination approche	Vaccination dose
1	Hyundong Jo	2021	0	3	/	*/*	Intramuscular	/
2	Hyundong Jo	2021	2	3	O	*Escherichia coli*	Intramuscular	0.015 mg
3	Hyundong Jo	2021	5	5	O	*Escherichia coli*	Intramuscular	0.15 mg
4	Hyundong Jo	2021	0	3	/	*/*	Intramuscular	/
5	Hyundong Jo	2021	2	3	A	*Escherichia coli*	Intramuscular	0.015 mg
6	Hyundong Jo	2021	5	5	A	*Escherichia coli*	Intramuscular	0.15 mg
7	Rodrigo Cañas-Arranz	2019	4	5	O	FMOC-solid	Intramuscular	2 mg
8	Rodrigo Cañas-Arranz	2019	4	5	O	FMOC-solid	Intramuscular	0.5 mg
9	Rodrigo Cañas-Arranz	2019	0	2	/	*/*	Intramuscular	/
10	Xiaoxiao Wang	2019	3	10	A	*Escherichia coli*	Intramuscular	1 mg
11	Xiaoxiao Wang	2019	0	3	/	*/*	Intramuscular	/
12	Xu Hai	2017	4	5	O	*T7*	Intramuscular	/
13	Xu Hai	2017	3	5	O		Intramuscular	0.5 mg
14	Xu Hai	2017	5	5	O	Inactivated vaccine	Intramuscular	/
15	Xu Hai	2017	0	2	/	*/*	Intramuscular	/
16	Yanmei Dong	2015	0	5	O	*/*	Intramuscular	/
17	Yanmei Dong	2015	4	5	O	Inactivated vaccine	Intramuscular	0.2 mg
18	Yanmei Dong	2015	1	5	O	*MS2 Phage*	Intramuscular	0.2 mg
19	Yanmei Dong	2015	3	5	O	*Escherichia coli*	Intramuscular	0.2 mg
20	Carolina Cubillos	2008	4	4	/	Synthesize	Intramuscular	1.4 mg
21	Carolina Cubillos	2008	0	2	/	*/*	Intramuscular	/
22	ChungDa Yang	2007	3	3	O	*/*	Intramuscular	5 mg
23	ChungDa Yang	2007	3	3	O	*/*	Intramuscular	1 mg
24	ChungDa Yang	2007	3	3	O	*/*	Intramuscular	0.5 mg
25	ChungDa Yang	2007	0	4	/	*/*	Intramuscular	/
26	Houhui Song	2005	8	10	O	*Benthamiana*	Intraperitoneal	0.1 mg
27	Houhui Song	2005	0	10	O	*/*	Intraperitoneal	/
28	Changyi Wang	2004	5	5	O	Synthesize	Intramuscular	2
29	Changyi Wang	2004	5	5	O	Synthesize	Intramuscular	1
30	Changyi Wang	2004	0	2	/	*/*	Intramuscular	/
31	Guangjin Li	2004	5	5	O	*Escherichia coli*	Intramuscular	0.8 mg
32	Guangjin Li	2004	5	5	O	*Escherichia coli*	Intramuscular	0.8 mg
33	Guangjin Li	2004	0	5	O	*/*	Intramuscular	/
34	JengHwan Wang	2003	8	8	O	*Escherichia coli*	Intramuscular	7 mg
35	JengHwan Wang	2003	0	2	/	*/*	Intramuscular	/
36	Ligang Wu	2003	3	3	O	*Tobacco*	Intramuscular	3 mg
37	Ligang Wu	2003	0	3	/	*Tobacco*	Intramuscular	/
38	EWC chan	2001	5	5	O	*Escherichia coli*	Intramuscular	2 mg
39	EWC chan	2001	2	5	O	*Escherichia coli*	Intramuscular	0.5 mg
40	EWC chan	2001	0	5	O	*Escherichia coli*	Intramuscular	1 mg
41	EWC chan	2001	5	5	O	Inactivated accine	Intramuscular	/
42	EWC chan	2001	0	5	/	*/*	Intramuscular	/
43	MA Kuprianova	2000	0	3	A	Synthesize	Intramuscular	1 mg
44	MA Kuprianova	2000	3	3	A	Synthesize	Intramuscular	2.5 mg

### Data synthesis

PPVDs of less than or equal to 1 mg provided protection against FMDV in 69.41% of pigs (Table 2). The OIE standard for FMDV vaccine was 75% (12/16). The data proved that the protection rate of PPVDs less than or equal to 1 mg was very low.

**Table 2:** Number of animals protected by PPVDs less than or equal to 1 mg group and the non-immune group.

	Author	Year	Non-immune group			PPVDs less than or equal to 1 mg		
			Protected pigs	Total pigs	Antigen payload	Protected pigs	Total pigs	Antigen payload
1	Hyundong Jo	2021	0	6	/	4	6	0.015 mg
2	Hyundong Jo	2021	0	6	/	10	10	0.15 mg
3	Rodrigo Cañas-Arranz	2019	0	2	/	4	5	0.5 mg
4	Xiaoxiao Wang	2019	0	3	/	3	10	1 mg
5	Xu Hai	2017	0	2	/	3	5	0.5 mg
6	Yanmei Dong	2015	0	5	/	4	10	0.2 mg
7	ChungDa Yang	2007	0	4	/	3	3	0.5 mg
8	ChungDa Yang	2007	0	4	/	3	3	1 mg
9	Houhui Song	2005	0	10	/	8	10	0.1 mg
10	Changyi Wang	2004	0	2	/	5	5	1 mg
11	Guangjin Li	2004	0	5	/	10	10	0.8 mg
12	EWC chan	2001	0	5	/	2	5	0.5 mg
	Total and ratio of protection		0	49	0%	59	85	69.41%

PPVDs greater than or equal to 2 mg group provided protection against FMDV in 97.22% (> 75%) pigs (Table. 3). This rate was acceptable. The protective rate of 2 mg or more group (97.22%) was higher than that of 1 mg or less group (69.41%).

**Table 3:** Number of animals protected by PPVDs greater than or equal to 2 mg group and the non-immune group.

	Author	Year	Non-immune group			PPVDs greater than or equal to 2 mg		
			Protected pigs	Total pigs	Antigen payload	Protected pigs	Total pigs	Antigen payload
1	Rodrigo Cañas-Arranz	2019	0	2	/	4	5	2 mg
2	ChungDa Yang	2007	0	4	/	3	3	5 mg
3	Changyi Wang	2004	0	2	/	5	5	2 mg
4	Ligang Wu	2003	0	3	/	3	3	3 mg
5	JengHwan Wang	2003	0	2	/	8	8	7 mg
6	EWC chan	2001	0	5	/	5	5	2 mg
	Total and ratio of protection		0	20	0%	35	36	97.22%

Table 4 shows that 93.75% of pigs were protected against FMDV by PPVDs group of 2 mg or more. The high dose protection rate was acceptable (> 75%). However, the low dose protective ratio was terrible. The protective rate (93.75%) of the group greater than or equal to 2 mg was higher than that of the group less than or equal to 1 mg (63.16% < 75%).

**Table 4:** Number of animals protected by PPVDs less than or equal to 1 mg group and PPVDs greater than or equal to 2 mg group.

	Author	Year	PPVDs less than or equal to 1 mg			PPVDs greater than or equal to 2 mg		
			Protected number	Total number	Antigen payload	Protected number	Total number	Antigen payload
1	Rodrigo Cañas-Arranz	2019	4	5	0.5 mg	4	5	2 mg
2	ChungDa Yang-A	2007	3	3	0.5 mg	3	3	5 mg
3	ChungDa Yang-B	2007	3	3	1 mg	3	3	5 mg
4	EWC chan	2001	2	5	0.5 mg	5	5	2 mg
5	MA Kuprianova	2000	0	3	1 mg	3	3	2.5 mg
	Total and ratio of protection		12	19	63.16%	15	16	93.75%

PPVDs group of less than or equal to 1 mg provides protection against FMDV in 56% of pigs, and the inactivated vaccine group provided protection against FMDV in 93.33% of pigs (Table 5). However, the group with PPVDs less than or equal to 1 mg (56%) was a terrible protection. The protective rate (56%) of the group less than or equal to 1 mg was lower than that of the inactivated vaccine group (93.33%). At present, there are only four experiments about the relationship between the PPVDs less than or equal to 1 mg group and inactivated vaccines.

**Table 5:** Number of animals protected by PPVDs less than or equal to 1 mg group and the inactivated vaccine group.

	Author	Year	Inactivated vaccine group			PPVDs less than or equal to 1 mg		
			Protected pigs	Total pigs	Antigen payload	Protected pigs	Total pigs	Antigen payload
1	Xu Hai	2017	5	5	/	3	5	0.5 mg
2	Yanmei Dong-A	2015	4	5	/	1	5	0.2 mg
3	Yanmei Dong-B	2015	4	5	/	3	5	0.2 mg
4	EWC chan	2001	5	5	/	2	5	0.5 mg
	Total and ratio of protection		14	15	93.33%	14	25	56%

## Discussion

In this study, mainly PPVDs were analyzed. In all selected studies, the pigs were immunized with polypeptide vaccines, and the dose used in the challenge experiments was within the approved range. However, it showed a difference between PPVDs ≤ 1 mg (63.16%) and PPVDs ≥ 2 mg (93.75%). The results also showed that the protective effects of the PPVDs ≤ 1 mg group (56%) did not reach the protective effect of the inactivated vaccines group (93.33%). It means that the antigen payload of polypeptide vaccines must have a prescribed standard. There are many ways to improve the protection provided by polypeptide vaccines^21^. For example, the vaccine can be prepared by linking it with a vector, which can increase the volume of antigen and help antigen-presenting cells recognize it^22^. In a study, the core polypeptide of the hepatitis B virus could be inserted with antigen^23^. In addition, when the epitope exists in the form of a dimer or polymer, the immunogenicity was stronger than that of a single epitope synthetic peptide^7^. An ideal PP vaccine should be a one-time immunization to prevent multiple serotypes of the FMDV, with long-term protection^24^. The PP vaccine also has many shortcomings. PP vaccine lacks sufficient stimulation of B cell epitopes, and carries fewer epitopes than inactivated vaccines^25^. The production cost of the PP vaccine is higher than the inactivated vaccine^26^.

There were many guidelines for doing meta-analysis^27,28^. The advantages of meta-analysis include a comprehensive retrieval strategy and qualification criteria for retrieval research. However, it must be acknowledged that there are some limitations in this meta-analysis. Firstly, the studies selected were only published in Chinese and English, which may restrict the inclusion of all other relevant studies on this subject. More languages can be combined to solve the problem. Secondly, the experimental and the control groups must be in the same document, there were only a limited number of documents. In the future, more data will be available to clarify the results. Thirdly, the expression vectors may affect the function of the polypeptide vaccine. With more and more data, classification and analysis can be considered according to the expression vectors in the future. Moreover, the application of statistical methods such as Mantel–Haenszel pooling and inverse variance method must conform to the normal distribution^29,30^ Due to the non-normal distribution data, we abandoned random effect meta-analysis to process the data^31,32^. Efthimiou published a meta-analysis guide for rare events, which is very suitable for the analysis of these data^33^. Due to zero event, we abandoned the forest plots^34,35^. As the data in Tables 4 and 5 are close to zero event meta-analysis, it is very misleading and dangerous to use I^2^ to measure heterogeneity. Because the inherent confidence interval is wide and I^2^ is small, the description and usage of I^2^ are given^36,37^. Although funnel chart is commonly used in meta-analysis, it is used to infer bias. However, our data is challenging, less than ten data, so it is not suitable for funnel chart^38^. The arcsine difference can also be used for data processing and comparison. This approach has been criticized for yielding non-interpretable summary results^39^. The first meta-analysis on FMD vaccine used a single scale meta-analysis to study the effect of FMD vaccine^40^. After that, some article performed meta-analyses related to FMDV too^41,42^. This analysis could guide future randomized controlled trials of higher quality to evaluate the effectiveness of polypeptide vaccines.

## Conclusion

Altogether, PPVDs has a dose-dependent protective effect on pigs and PPVDs ≤ 1 mg group was lower than the inactivated vaccines group. In order to establish a clear conclusion on the immune response of polypeptides, future randomized controlled trials need to be designed with more data and long-term field and experimental animal studies. Although there are some shortcomings in this research, the epidemiological policies should pay enough attention to it.

## Data Availability

All data generated or analyzed during this study are included in this published article [and its supplementary information files].

## References

[1] Zhao FR, Xie YL, Liu ZZ, Shao JJ, Li SF, Zhang YG, Chang HY (2017). Transcriptomic analysis of porcine PBMCs in response to FMDV infection. Acta Trop..

[2] Dong YM, Zhang GG, Huang XJ, Chen L, Chen HT (2015). Promising MS2 mediated virus-like particle vaccine against foot-and-mouth disease. Antiviral Res..

[3] Brown F (1988). Use of peptides for immunization against foot-and-mouth disease. Vaccine.

[4] Baranowski E, Ruiz-Jarabo CM, Lim F, Domingo E (2001). Foot-and-mouth disease virus lacking the VP1 G-H loop: the mutant spectrum uncovers interactions among antigenic sites for fitness gain. Virology.

[5] Fang M (2012). Correlation between efficacy and structure of recombinant epitope vaccines against bovine type O foot and mouth disease virus. Biotechnol. Lett..

[6] Hewat EA (1997). Structure of the complex of an Fab fragment of a neutralizing antibody with foot-and-mouth disease virus: positioning of a highly mobile antigenic loop. EMBO J..

[7] Kupriianova MA, Zhmak MN, Koroev DO, Chepurkin AV, Ivanov VT (2000). Synthetic peptide designs based on immunoactive fragments of the VP1 protein of the foot-and-mouth disease virus strain A22. Bioorg. Khim..

[8] Chan EW (2000). An immunoglobulin G based chimeric protein induced foot-and-mouth disease specific immune response in swine. Vaccine.

[9] Yang CD (2007). Induction of protective immunity in swine by recombinant bamboo mosaic virus expressing foot-and-mouth disease virus epitopes. BMC Biotechnol..

[10] Caas-Arranz R, Forner M, Defaus S, León PD, Andreu D (2020). A single dose of dendrimer B2T peptide vaccine partially protects pigs against foot-and-mouth disease virus infection. Vaccines.

[11] Jo H (2021). The HSP70-fused foot-and-mouth disease epitope elicits cellular and humoral immunity and drives broad-spectrum protective efficacy. NPJ Vaccines.

[12] Valanzano A (2004). Rules of good practice in the care of laboratory animals used in biomedical research. Ann. dell'Istituto Super. Sanita.

[13] Wang XX, Sun P, Jia HJ (2019). Prokaryotic expression and immune effect of major antigenic epitope regions of type A foot-and-mouth disease virus capsid protein. Chin. Vet. Sci..

[14] Hai X, Xi B, Yu L, Liu Y, Hou J (2017). Immunogenicity of T7 bacteriophage nanoparticles displaying G-H loop of foot-and-mouth disease virus (FMDV). Vet. Microbiol..

[15] Cubillos C (2008). Enhanced mucosal immunoglobulin A response and solid protection against foot-and-mouth disease virus challenge induced by a novel dendrimeric peptide. J. Virol..

[16] Song H (2005). A novel mucosal vaccine against foot-and-mouth disease virus induces protection in mice and swine. Biotechnol. Lett..

[17] Li G (2004). Comparison of immune responses against foot-and-mouth disease virus induced by fusion proteins using the swine IgG heavy chain constant region or beta-galactosidase as a carrier of immunogenic epitopes. Virology.

[18] Wang JH (2003). Induction of immunity in swine by purified recombinant VP1 of foot-and-mouth disease virus. Vaccine.

[19] Wu L (2003). Expression of foot-and-mouth disease virus epitopes in tobacco by a tobacco mosaic virus-based vector. Vaccine.

[20] Kuprianova MA (2000). Synthetic peptide constructs on the basis of immunoactive fragments of the A22 Strain VP1 of the foot-and-mouth disease virus. Rus. J. Bioorg. Chem..

[21] Dietrich, J., Ljunghall, S. & Sjogren, S. Methods useful in the treatment of bone resorption diseases. (2014).

[22] Shanmugaraj B, Khorattanakulchai N, Phoolcharoen W (2022). SARS-CoV-2 variants: a continuing threat to global health. Asian Pac. J. Trop. Med..

[23] Shiau AL, Murray K (2015). Mutated epitopes of hepatitis B surface antigen fused to the core antigen of the virus induce antibodies that react with the native surface antigen. J. Med. Virol..

[24] Dias C, Moraes MP, Segundo DS, Teresa D, Grubman MJ (2011). Porcine type I interferon rapidly protects swine against challenge with multiple serotypes of foot-and-mouth disease virus. J. Interferon Cytokine Res. Off. J. Int. Soc. Interferon Cytokine Res..

[25] Ahmad S, Shahid F, Qamar M, Rehman HU, Saeed H (2021). Immuno-informatics analysis of pakistan-based HCV subtype-3a for chimeric polypeptide vaccine design. Vaccines.

[26] Joensuu JJ, Niklander-Teeri V, Brandle JE (2008). Transgenic plants for animal health: plant-made vaccine antigens for animal infectious disease control. Phytochem. Rev..

[27] Higgins J, Green S (2011). GSe, cochrane handbook for systematic reviews of interventions. Naunyn. Schmiedebergs Arch. Exp. Pathol. Pharmakol..

[28] Mikolajewicz N, Komarova SV (2019). Meta-analytic methodology for basic research: a practical guide. Front. Physiol..

[29] Jackson D, White IR (2018). When should meta-analysis avoid making hidden normality assumptions. Biom. J..

[30] Jackson D, Law M, Stijnen T, Viechtbauer W, White IR (2018). A comparison of seven random-effects models for meta-analyses that estimate the summary odds ratio. Stat. Med..

[31] Higgins JP (2008). Commentary: Heterogeneity in meta-analysis should be expected and appropriately quantified. Int. J. Epidemiol..

[32] Borenstein M, Hedges LV, Higgins JP, Rothstein HR (2010). A basic introduction to fixed-effect and random-effects models for meta-analysis. Res. Synth. Methods.

[33] Efthimiou O (2018). Practical guide to the meta-analysis of rare events. Evid. Based Ment. Health.

[34] Bradburn MJ, Deeks JJ, Berlin JA, Russell Localio A (2007). Much ado about nothing: a comparison of the performance of meta-analytical methods with rare events. Stat. Med..

[35] Sweeting MJ, Sutton AJ, Lambert PC (2004). What to add to nothing? Use and avoidance of continuity corrections in meta-analysis of sparse data. Stat. Med..

[36] Rücker G, Schwarzer G, Carpenter JR, Schumacher M (2008). Undue reliance on I(2) in assessing heterogeneity may mislead. BMC Med. Res. Methodol..

[37] Borenstein M, Higgins JP, Hedges LV, Rothstein HR (2017). Basics of meta-analysis: I(2) is not an absolute measure of heterogeneity. Res. Synth. Methods.

[38] Egger M, Davey Smith G, Schneider M, Minder C (1997). Bias in meta-analysis detected by a simple, graphical test. BMJ.

[39] Rücker G, Schwarzer G, Carpenter J, Olkin I (2009). Why add anything to nothing? The arcsine difference as a measure of treatment effect in meta-analysis with zero cells. Stat. Med..

[40] Halasa T, Boklund A, Cox S, EnøE C (2011). Meta-analysis on the efficacy of foot-and-mouth disease emergency vaccination. Prev. Vet. Med..

[41] Eblé PL, Koeijer A, Jong M, Engel B, Dekker A (2008). A meta-analysis quantifying transmission parameters of FMDV strain O Taiwan among non-vaccinated and vaccinated pigs. Prev. Vet. Med..

[42] Mardones F, Perez A, Sanchez J, Alkhamis M, Carpenter T (2010). Parameterization of the duration of infection stages of serotype O foot-and-mouth disease virus: an analytical review and meta-analysis with application to simulation models. Vet. Res..

